# Differences in isotretinoin start, interruption, and early termination across race and sex in the iPLEDGE era

**DOI:** 10.1371/journal.pone.0210445

**Published:** 2019-03-26

**Authors:** Alexandra Charrow, Fan Di Xia, Jessica Lu, Michael Waul, Cara Joyce, Arash Mostaghimi

**Affiliations:** 1 Harvard Combined Dermatology Residency Training Program, Brigham & Women’s Hospital, Harvard Medical School, Boston, Massachusetts, United States of America; 2 Harvard Medical School, Boston, Massachusetts, United States of America; 3 McGill Medical School, Montreal, Quebec, Canada; 4 Department of Public Health Sciences, Loyola University, Chicago, Illinois, United States of America; 5 Department of Dermatology, Brigham & Women’s Hospital, Harvard Medical School, Boston, Massachusetts, United States of America; University of Manitoba, CANADA

## Abstract

**Background:**

iPLEDGE is the mandatory regulatory program for isotretinoin in the United States, aimed to prevent isotretinoin-related teratogenicity. However, little is known about potential unintended impact of the program, including delay in isotretinoin initiation, course interruption, and premature termination, which may vary across sex and racial domains.

**Objective:**

To determine whether differences in isotretinoin start, interruption, and completion exist across sex and racial domains and whether iPLEDGE regulations contribute to such differences.

**Methods:**

Retrospective review of isotretinoin courses of patients prescribed isotretinoin for acne at the Brigham & Women’s Hospital and Massachusetts General Hospital from 2008–2016.

**Results:**

**418** patients were included in analysis after being tightly matched across age and gender. 43.5% of non-white patients ended their course early compared to 30.1% of white patients (p = 0.010). iPLEDGE -related barriers were the most commonly specified reasons for delayed starting and interruption.

**Conclusion:**

iPLEDGE may disproportionately contribute to access barriers for non-white patients. Continued evaluation of iPLEDGE is needed to minimize unintended barriers to access.

## Introduction

Isotretinoin is a highly effective medication prescribed for the treatment of recalcitrant or severe cystic and nodular acne [1]. Timely initiation of the drug can offer life-long benefits to those with deforming or scarring disease. Its efficacy, however, is tempered by a side-effect profile that includes severe teratogenicity [2]. Since the drug’s approval in 1981, several government regulatory strategies have been developed to prevent pregnancy and reduce adverse outcomes in patients prescribed isotretinoin, the most recent iteration of which in the United States is iPLEDGE [3].

iPLEDGE was implemented in 2006 and specifies requirements for both the isotretinoin prescriber and recipient prior to pharmacy dispensation. At initiation, patients, physicians, pharmacists and drug suppliers must be registered within the iPLEDGE database and document and review a standardized information packet regarding the medication, its side effects, and its teratogenicity. Female patients must report 2 forms of effective contraception starting at least 1 month prior to and through 1 month after the isotretinoin course. In addition, each month the prescribing physician must confirm counselling, the use of two contraceptives, and negative pregnancy testing for all female patients within a 7-day window of testing. Following prescriber confirmation, the patient must confirm two methods of contraception within the same 7-day window. After this step the registered pharmacy can fill the prescription.

Male patients and post-menopausal or surgically infertile women must undergo the same counselling, monthly registration, and physician and pharmacy attestation but their prescribing window is 30 days [4]. If any of these steps are missed or not completed within the required timeframe, isotretinoin cannot be dispensed.

The iPLEDGE registry has been controversial throughout its existence. The registry has not been demonstrated to decrease rates of pregnancy among patients taking isotretinoin as compared to proceeding regulatory systems [5]. Concerns about the use of iPLEDGE by transgendered males and women who have sex with women have been raised as iPLEDGE does not provide a registration option that both captures their gender or sexual identity and their pregnancy potential [6].

Data from other regulatory programs for isotretinoin suggest that these programs may also promote healthcare disparities. Under the preceding regulatory strategies to iPLEDGE, isotretinoin was under-prescribed to African American patients and women as compared to white patients and men [7]. In New Zealand, where oral isotretinoin can be prescribed only by dermatologists registered within a governmental database, ethnic minorities such as the Maori and Pacific people are less likely to be prescribed isotretinoin than their white counterparts though it is not known if this disparity is due to the regulatory structure or other factors [8]. The impact of iPLEDGE on patient access to medications has not been evaluated.

In this study, we evaluate differences in isotretinoin start, interruption, and course completion across sex and race in the context of the US iPLEDGE program. We hypothesize that the restrictive nature of the iPLEDGE regulation will disproportionately affect women and non-white individuals leading sub-optimal treatment of acne.

## Methods

This project was approved by the Partners Healthcare Human Research Committee Institutional Review Board. Given the retrospective nature of this review, informed consent was waived. We queried all medical records from January 1, 2008 through June 1, 2016 at Brigham & Women’s Hospital, Massachusetts General Hospital, and affiliated clinics using the Research Patient Data Repository (RPDR), a clinical data registry that captures clinical information on all patients seen at Partners Healthcare. Our search parameters identified non-white patients prescribed isotretinoin for the treatment of acne and who initiated their first treatment after January 1, 2008. A cohort of white patients was identified through matching to the non-white sample on age at isotretinoin initiation (+/- 1 year), sex, and date of first isotretinoin course (+/- 1 year). Of this sample, we excluded patients who were previously prescribed isotretinoin, who were prescribed isotretinoin for a non-acne indication, who were prescribed isotretinoin entirely or partially outside of the Partners system, who initiated their isotretinoin course prior to January 1, 2008, or who transferred their care to or from an out-of-network physician.

Identified electronic medical records were individually reviewed (by A.C., F.D.X., J.L., and M.W.) and data were extracted along the following parameters: patient demographics including sex, race (African American or Black, Asian, white, American Indian, Native Hawaiian, 2 or more races, other, or unknown), ethnicity (Hispanic, not Hispanic, or unknown), primary language (English, Spanish, or other), insurance type at treatment initiation (private, public, none), weight at isotretinoin initiation, and patient portal access. Because Hispanic vs not Hispanic ethnicity is consistently recorded in the medical record and other ethnicities are entered with variable consistency and specificity, only Hispanic ethnicity was collected. Documented nursing and physician communications were reviewed to determine medication utilization including total mg/kg isotretinoin dosage achieved, number of months prescribed, delays in initiation of treatment, treatment interruptions, and early terminations, and the reasons documented for the above. Optimal weight-based dosing was considered 120 mg/kg based on current dermatologic guidelines [9].

Delays in initiation were defined as documented physician, nursing, or patient communication indicating missed pick-up, missed laboratory tests, missed appointment or delay for any reason leading to delay in initiation of greater than 3 days. The required 30-day period of contraception for women between two negative pregnancy tests prior to initiation was not defined as a “delay” for this study. Reasons for delay excluded the acquisition of pregnancy testing required of all female patients but included missed pregnancy testing or missed laboratory acquisition. Interruptions in course were defined as physician, nursing, or patient documentation of an interruption in taking the medication for 28 days or greater with resumption of the medication subsequently without indication of starting of a new cycle. Early termination was defined as cases whereby physician notes indicated intention to continue the medication the following month but the medication was not continued or no further notes for the patient were documented for at least 1 year.

The first 10% of each reviewer’s data entry were independently validated by the managing reviewer (JL) to ensure consistency between reviewers. Study data were collected and managed using REDCap (Research Electronic Data Capture) electronic data capture tool hosted through Partners Healthcare [10].

### Statistical analysis

Descriptive statistics on patient characteristics and reasons for delayed start, interruptions, and early termination were presented to describe the study cohort after exclusion criteria were applied. Rates of delayed starts, interruptions, early terminations, and sub-optimal dosages were compared for matched pairs using McNemar’s tests. All data analysis was performed using statistical software SAS 9.4 (SAS Institute, Cary, NC).

## Results

### Patient characteristics

A total of 1108 matched patients were identified, of whom 418 were included for analysis. 262 individuals were excluded as they were prescribed isotretinoin partially or fully outside of the Partners system, 57 as they were prescribed isotretinoin for a non-acne indication, 107 as they had previously been prescribed isotretinoin, and 19 individuals were removed as they were missing data regarding total medication dosage achieved. Of the 663 patients who remained, 418 individuals could be tightly matched for analysis. In the analytic cohort, the average age at initiation of isotretinoin was 28.3 ± 7.4 years. 53.6% of the cohort was female, 50.0% identified as white, 15.1% as Black/African American, 14.4% as Asian and 19.9% as Hispanic. 20.2% of the cohort had public insurance (Table 1).

**Table 1:** Patient characteristics.

	Overall (n = 418)	White n = 209 (50.0)	Non-white n = 209 (50.0)
Age, mean (SD)	28.3 (7.4)	28.2 (7.3)	28.3 (7.4)
Female, n (%)	224 (53.6)	112 (53.6)	112 (53.6)
Race, n (%)			
White	209 (50.0)	209 (100.0)	0 (0.0)
Black	63 (15.1)	0 (0.0)	63 (30.1)
Hispanic	83 (19.9)	0 (0.0)	83 (39.7)
Asian	60 (14.4)	0 (0.0)	60 (28.7)
American Indian or Alaskan Native	3 (0.7)	0 (0.0)	3 (1.4)
Insurance, n (%)			
Private	311 (75.7)	178 (86.8)	133 (64.6)
Public	83 (20.2)	21 (10.2)	62 (30.1)
None	17 (4.1)	6 (2.9)	11 (5.3)

### Gaps in treatment

Of the 418 patients included in the analysis, 9.1% had a delay in starting isotretinoin, 8.4% had unplanned interruptions in treatment, and 36.8% had early termination of their course. The most commonly specified reasons were delay were iPLEDGE-related reasons including computer issues navigating the iPLEDGE website (n = 11, 28.9%), missed pick-up windows (n = 6, 15.8%), and missed/delayed MD appointment/blood tests (n = 7, 18.4%). iPLEDGE-related reasons including missed/delayed appointments/tests (n = 9, 25.7%), missed pick-up windows (n = 8, 22.9%), and computer issues with iPLEDGE (n = 2, 5.7%) were also cumulatively the most common reasons for interruptions. Loss to follow-up was the most common reason for early termination (n = 82, 53.2%), followed by inability to tolerate medication (n = 33, 21.4%) (Table 2).

**Table 2:** Reasons for delayed start, interruptions, and early terminations.

Delayed in starting, n (%)	38 (9.1)
Reason for delay	
Missed/delayed appointments/tests	7 (18.4)
Missed pick-up window	6 (15.8)
Computer issues with iPLEDGE	11 (28.9)
Pregnancy	0 (0.0)
Other	15 (39.5)
Unspecified	5 (13.2)
Interruptions after starting, n(%)	35 (8.4)
Reason for interruptions	
Missed/delayed appointments/tests	9 (25.7)
Missed pick-up window	8 (22.9)
Computer issues with iPLEDGE	2 (5.7)
Side effects	8 (22.9)
Pregnancy	1 (2.9)
Other	13 (37.1)
Unspecified	1 (2.9)
Early termination, n (%)	154 (36.8)
Reason for early termination	
Lost to follow-up or unknown	82 (53.2)
Missed pick-up window	2 (1.3)
Computer issues with iPLEDGE	0 (0.0)
Side effects/poorly tolerated	33 (21.4)
Patient personal preference	19 (12.3)
Other	18 (11.7)

### Impact of race

**Fig 1:**
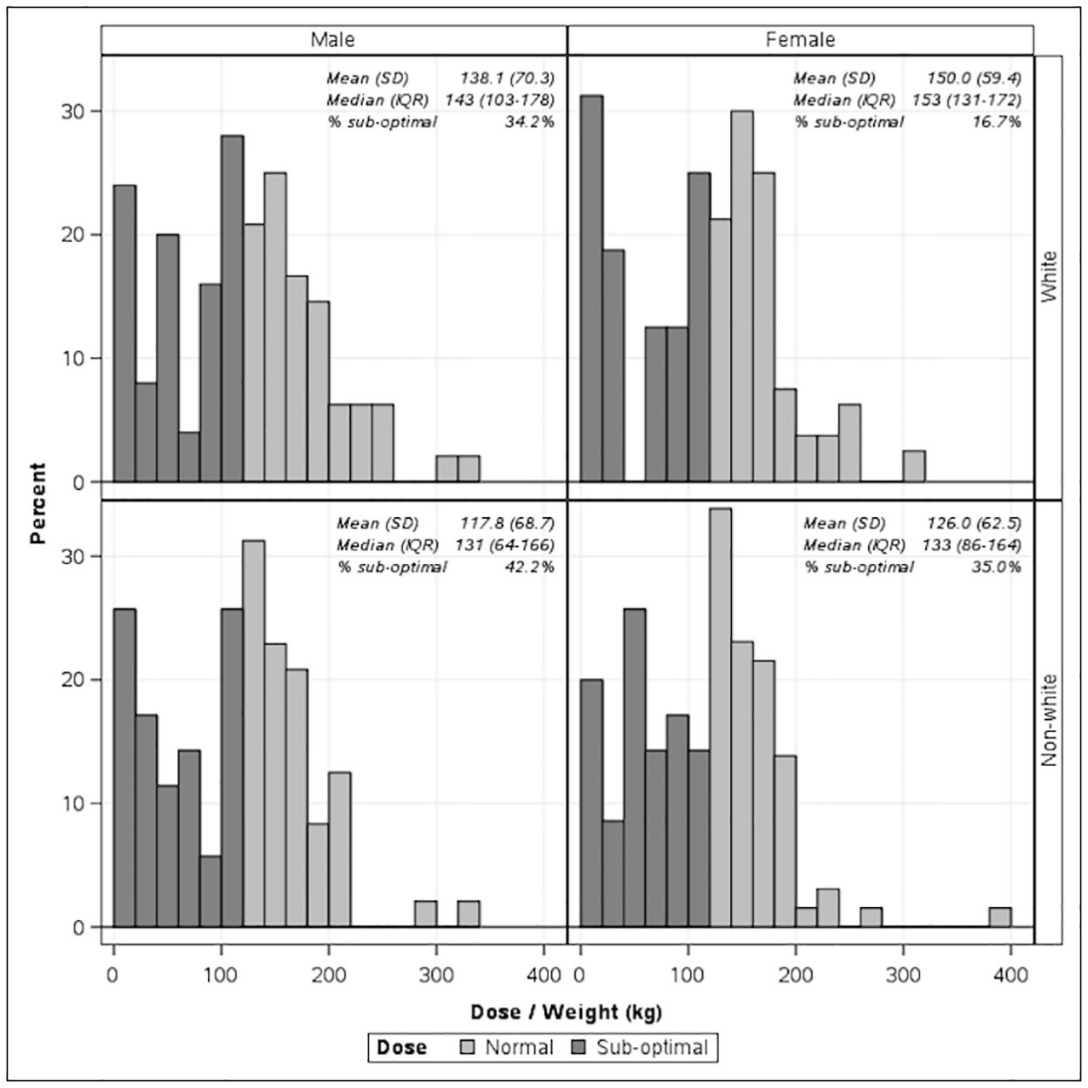
Cumulative dose of isotretinoin achieved by race and sex.

43.5% of non-white patients ended their course early compared to 30.1% of white patients (p = 0.010). Additionally, 12.0% of non-white patients experienced interruptions in treatment course compared to 4.8% of white patients (p = 0.017). Race was not associated with a delay in medication initiation (p = 0.99) (Table 3). Non-white patients were less likely to reach a goal cumulative dose of > 120 mg/kg, with 42.2% non-white males reaching a sub-optimal cumulative dose as compared to 34.2% white males. 35.0% of non-white females reached a sub-optimal dose as compared to 16.7% of white females (Fig 1).

**Table 3:** Delayed starts, interruptions, and early terminations among matched pairs.

	Delayed Start, n (%)	p-value	Interruptions, n (%)	p-value	Early termination, n (%)	p-value
White	19 (9.1)	0.99	10 (4.8)	0.017	63 (30.1)	0.010
Non-white	19 (9.1)		25 (12.0)		91 (43.5)	

## Discussion

This study demonstrates differences in delayed starting, interruption, and early termination of isotretinoin treatment course across patients of different race. Non-white patients were more likely than white patients to experience medication interruptions and early terminations and were more likely to achieve sub-optimal doses of isotretinoin.

The most common reasons cited for delays and interruptions were related to the logistics of the iPLEDGE requirements, including computer issues, missed pick-up windows, and missed/delayed appointments/tests. These findings suggest that differences in delayed initiation and medication interruption are in part related to iPLEDGE regulation requirements including time-mandated monthly visits, sensitively-timed laboratory testing, and the technological interface. Despite efforts to reduce barriers to iPLEDGE, patients with lower incomes and non-white patients demonstrate lower comfort with and utilization of online patient portals [11, 12], suggesting that significant electronic requirements may serve as barriers to access for such populations. Similar limitations exist for reliable access to telephones and mobile devices [13].

This work adds to existing data demonstrating an under-prescribing of isotretinoin to female and non-white patient [6,7]. The regulation of isotretinoin is one of only a handful of teratogenic medications with such formalized and extensive governmental regulations. Methotrexate, and many antiepileptic medication, are highly teratogenic but are prescribed to young people for rheumatologic conditions, other dermatologic conditions, and epilepsy without a similar regulatory infrastructure. While the objective of iPLEDGE regulations are intended to both educate patients on isotretinoin side effects and teratogenicity, and prevent isotretinoin-associated-teratogenicity, it is important to minimize unintended access barriers and identify less restrictive, more effective models for regulation that reduce pressures on patients and providers.

To date, studies have not been able to demonstrate that iPLEDGE is more effective at reducing isotretinoin-related pregnancies as compared to the preceding regulatory structure [5]. This, coupled with evidence demonstrating differences in prescription, start, and maintenance of the medication across demographic domains, force us to reconsider whether iPLEDGE’s benefits such as patient education are outweighed by its unintended effects.

Our findings must be interpreted within the context of our study design. This study was conducted in a single metropolitan area and may be limited in generalizability to the rest of the nation. Minority and socioeconomic status may be more strongly correlated in the greater Boston area than in other parts of the nation [14]. Regions with different patient demographics may have specific systems in place to ensure patient retention, computer, and health literacy before initiation of isotretinoin. Our study also lacks a control group; it is possible that challenges surrounding adherence may be associated with an unidentified confounder. Moreover, while the most common reasons cited for delays and interruptions were related to the logistics of the iPLEDGE requirements, without a control group, it is difficult to know if disparities are purely due to iPLEDGE regulation themselves.

## Conclusions

Our study demonstrates that differences in delayed start, interruption, and premature termination of isotretinoin exist across racial domains. These findings suggest that the most common reasons for delayed start and interruption of isotretinoin were associated with iPLEDGE regulations themselves, suggesting that such regulations affect the timely start and uninterrupted use of isotretinoin for patients from different backgrounds. While the goal of reducing teratogenicity is necessary and critical, access to isotretinoin for severe, recalcitrant acne across all demographic domains is important. Our study calls for continued evaluation and reform of iPLEDGE to minimize unintended barriers to access.
